# Measures of the Constitutive Immune System Are Linked to Diet and Roosting Habits of Neotropical Bats

**DOI:** 10.1371/journal.pone.0054023

**Published:** 2013-01-14

**Authors:** Karin Schneeberger, Gábor Á. Czirják, Christian C. Voigt

**Affiliations:** 1 Leibniz Institute for Zoo and Wildlife Research, Berlin, Germany; 2 Freie Universität Berlin, Department Animal Behaviour, Berlin, Germany; University of Georgia, United States of America

## Abstract

Ecological and social factors are central in the emergence and transmission of infectious diseases, thus bearing the potential for shaping a species’ immune functions. Although previous studies demonstrated a link between social factors and the cellular immune system for captive mammals, it is yet poorly understood how ecological factors are connected with the different branches of the immune system in wild populations. Here, we tested how variation in aspects of the constitutive cellular and humoral immune system of free ranging bats is associated with two ecological factors that likely influence the putative risk of species to become infected by parasites and pathogens: diet and shelter. We found that white blood cell counts of 24 syntopic Neotropical bat species varied with the species’ diet and body mass. Bats that included at least partially vertebrates in their diet exhibited the highest white blood cell counts, followed by phytophagous and insectivorous species, which is in agreement with the assumption that the immune system varies with the pathogen transmission risk of a trophic level. The soluble part of the constitutive immune response, assessed by an *in vitro* bacterial killing assay, decreased with increasing roost permanence. Our results suggest that the ecology is an important factor in the evolution of the immune system in bats and probably also other mammals.

## Introduction

The immune system provides animals with a set of cellular and molecular defence mechanisms against potentially infectious agents. Parasites and pathogens impose strong selective pressure on their hosts by reducing, for example, host fitness [1]. Social and ecological factors that are linked to parasite and pathogen transmission risks are therefore most likely influencing the immune system of mammals. Previous studies have shown that high population densities and large group sizes of hosts may increase the risk for horizontal transmission of pathogens due to frequent encounters between infected and healthy individuals [2]–[4]. For example, pathogen prevalence increases with increasing group size in both birds and mammals [5]–[8]. Similarly, ecological factors such as feeding habits have been shown to influence the pathogen transmission risk when vertebrates are likely to ingest contaminated food or infected prey [9], [10]. Therefore, both social and ecological factors may affect pathogen transmission risk among animals, and as a consequence the immune competence of animals should be related to these factors. Hosts with a better immune defence are expected to suffer less from pathogens, as has been shown in barn swallows (*Hirundo rustica*), where individuals with a stronger immune response survive better than less immune competent individuals [11]. However, as mounting, using and maintaining an effective immune response is energetically costly [12], individuals may have to trade these costs against other life-history components such as growth and reproductive output [13]. Therefore, the relative threat of infection resulting from the putative pathogen transmission risk of an ecological niche should be mirrored by the immune competence: species with a low infection risk should maintain a relatively low basal immune competence compared to species with high infection risk in order to minimise physiological and behavioural trade-offs.

Nunn and colleagues were the first to investigate social and ecological factors influencing the immune system of mammals by comparing the cellular constitutive immune system among healthy captive primates and carnivores. They showed that promiscuous species have higher white blood cell counts than monogamous species, probably due to an increased risk of acquiring sexually transmitted diseases [14]–[16]. However, other factors such as group size and diet failed to show general correlations with the cellular immune system [15], [16]. Only eosinophils increased with the percentage of meat in the diet of carnivores [16]. Additionally, while Nunn and colleagues used data obtained from healthy captive wildlife species, inter-specific studies on free-ranging mammals regarding the influence of ecological factors on the immune system are still lacking. In contrast to free-ranging animals, captive animal populations may face a lowered disease risk when managed by veterinarians and nutritional experts. Furthermore, while the cellular aspect of the constitutive immune system has already been investigated [15], [16], studies on the soluble immune factors are restricted to intra-specific studies only (e.g. [17], [18]).

Here, we investigated aspects of both the cellular and soluble part of the constitutive immune system of free-ranging mammals and ask whether their variation is associated with ecological factors. Bats are an ideal study group to investigate these questions, as they not only occupy numerous ecological niches, but are also associated with a number of emerging infectious diseases [19], [20], including the fungus *Geomyces destructans,* the causative agent of the White-Nose Syndrome, which recently lead to dramatic declines in North American bat populations [21]. Specific mechanisms of disease transmission are poorly understood in bats, yet some studies suggest horizontal transmission of pathogens via contaminated food. For example, Nipah viruses are probably transmitted via fruits contaminated by faeces, urine or saliva of frugivorous bats [22], [23], and rabies viruses are known to be transmitted when sanguinivorous vampire bats feed on their prey [24]. Bats cover a wide range of trophic levels, ranging from nectarivory, frugivory, omnivory, insectivory to carnivory. Trophic levels may vary with respect to their pathogen transmission risk according to the likelihood of animals to consume food items contaminated with infective agents and parasites. We therefore expected that feeding habits of bats influence their immune system according to the relative threat of infection with bacteria, viruses and parasites by oral-faecal route or by direct contact. Sanguinivorous species and species feeding at least partially on vertebrates (hereafter called carnivorous bats) face pathogen transmission risk due to direct contact with closely related prey species, while phytophagous species (frugivores and nectarivores) may feed on food sources that are potentially contaminated by faeces and saliva. Insects are important intermediate hosts for pathogens such as haemoparasites, different bacterial and viral agents, which may as well impose a selective pressure on the immune system of insectivorous bats. We therefore hypothesise that investment in the cellular immune system and the bacterial killing ability, as a measure of the constitutive innate immune system, will vary between species of different trophic levels.

Besides their diversity in dietary niches, bats also use a variety of structures as daytime shelters [25]. Patterson and colleagues [26] found that bat species roosting in more permanent and protected sites have a higher ectoparasite load than species using more ephemeral structures, highlighting that shelters vary in pathogen transmission risk. Furthermore, bacteria, viruses and parasites may be more abundant in roosts that are well protected from environmental factors such as precipitation. Thus, bats may face an increased risk of infection when using sheltered structures. Accordingly, we hypothesise that immune function should co-vary with shelter type. Therefore, we predict that species roosting in more permanent sites, presumably with higher risk of infection, would exhibit greater investment in the cellular and soluble parts of the constitutive immune system as compared with species roosting in more ephemeral structures.

In this study, we investigated aspects of both the cellular and soluble part of the constitutive immune system of 24 free-ranging Neotropical bat species. We used blood smears to estimate number of total and differential WBC counts, a method which has been used in birds and bats before [27], [28]. WBC count is characteristic for cell-mediated processes in response to infections and can been used as an indirect measure of an individual’s investment in cellular immune defence. Additionally, we extracted plasma from each individual for measuring the *in vitro* bacterial killing activity (BKA) mediated by the complement and other antibacterial proteins. Thus, we obtained quantitative measurements of two major parts of the constitutive immune system of each individual, which can then be averaged on the species level and compared to the mean of other species with respect to diet and shelter permanence and protection.

## Materials and Methods

### Ethic Statement

This study was approved by the institutional animal welfare and ethics committee of the Leibniz Institute for Zoo and Wildlife Research (permit number: 2011-08-01). Sample collection was authorised by the Ministerio del Ambiente y Energia (MINAE; permit number 163-20911-SINAC) of Costa Rica and complied with the current laws of the country.

### Blood Sampling

We collected samples from 178 individuals belonging to 24 bat species at “La Selva” Biological Station (10°25′N; 84°00′W, Province Heredia, Costa Rica) in November and December 2010 by catching bats between 5 pm and 10 pm at ground level using nylon mist nets (2.5 m height, Ecotone, Gdynia, Poland). Species were identified according to Timm and LaVal [29]. As it is not possible to distinguish between *Artibeus phaeotis* and *Artibeus watsoni* in the field, we will refer to these two species as *Artibeus watsoni c.f.* [30]. *Saccopteryx bilineata* were caught at dawn (5 am–7 am) when individuals returned to their daytime roost. Captured individuals were weighed with a spring balance (accuracy 0.5 g, Pesola balance; Switzerland). Sex, age and reproductive status were assessed. Juveniles were distinguished from adults by examining the degree of the epiphysial closure of the phalanges. Pregnant and lactating bats as well as juveniles were releases immediately at the site of capture. From all other bats, a small blood sample of no more than 5% of the total blood volume was taken by punctuating the antebrachial vein with a sterile needle and collecting the blood droplets with a heparinised capillary. A subsample of the blood was taken to prepare a blood smear on glass slides (Microscope Slides (76×26 mm), cut edges, Menzel, 38116 Braunschweig, Germany). The plasma was collected after centrifugation and stored at −80°C until further analysis. All bats were released at the site of capture.

### White Blood Cell Counts

Blood smears were stained with May-Gruenwald’s solution (#T863.2, Carl Roth GmbH) and Giemsa (#T862.1, Carl Roth GmbH). Total WBC count for each individual was estimated manually by taking the mean of 10 visual fields, counting the cells with a microscope under 200× magnifications. Some former publications using the same method multiplied this mean with a certain species-specific constant to obtain the number of white blood cells per microliter (e.g. [31], [32]). As no such constant is known for bats yet, and multiplication would not change the relative differences among species, we used the mean number of leukocytes per visual field for statistical analysis.

Additionally, after estimating the total WBC counts from blood smears, we validated this method by checking for correlation between our data obtained from blood smears and data obtained with a conventional method (Unopette™ capillary system) of the same species at the same site in a previous year [30], finding a strong positive correlation (linear model; R^2^ = 0.803; N = 12; t = 7.06; p<0.001).

Differential white blood cell (DWBC) counts were performed counting 100 leukocytes under 1000× magnification (oil immersion) and calculating the relative numbers of lymphocytes, monocytes, neutrophils, basophils and eosinophils. Absolute numbers of the different leukocytes were calculated by multiplication with total WBC counts.

### Bacterial Killing Activity

We measured the soluble aspect of the constitutive innate immunity by assessing the *in vitro* bacterial killing activity of the plasma against *Escherichia coli* [33] following the method of Tieleman and colleagues [34]. This assay has been used previously on different free-ranging and captive wild species, including bats [17], [33], [35]. Plasma samples were diluted 1∶20 in CO_2_-independent media (#18045, Gibco-Invitrogen, CA), enriched with 4 mM L-Glutamine (#25030, Gibco-Invitrogen, CA) and 5% Fetal Calf Serum (#S0115, Biochrom AG). To each diluted sample (140 µl) we added 10 µl of a suspension of live *E. coli* (ATCC #8739). The bacterial suspension was adjusted to a concentration of ∼200 colonies per 50 µl of diluted plasma-bacteria mixture. After incubation, for 30 min at 37°C (mammalian body temperature), 50 µl of the plasma-bacteria mixture was spread aliquots onto Tryptic Soy Agar plates (#CP70.1, Carl Roth GmbH) in duplicate, and the plates were incubated overnight at 37°C. To obtain the initial number of bacteria that we had before starting to interact with the plasma, we diluted 140 µl media alone with bacterial suspension and plated immediately. On the following day the colony-forming units were counted and the bacterial killing activity was defined as the percent of the killed bacteria, which was calculated as 1– (average of the viable bacteria after incubation/the initial number of bacteria). The average was calculated from two plates per sample.

Storage time of the samples can have an influence on BKA [36]. The storage time of our samples ranged from 41 to 81 days, with a mean of 63 days. We therefore tested the effect of storage time on BKA, and could not find an effect (Spearman rank correlation; rho = −0.062; p = 0.442; n = 158). Also, we use species mean for our analysis and as individuals of one species were not all captured nor analysed on the same day, the mean storage time of the samples of one species should be approximately the same. We tested this assumption and found no evidence against it, as there was no correlation between mean storage time and mean BKA of the species (Spearman rank correlation; rho = −0.120; p = 0.576; n = 24).

### Data Sources from Literature and Statistical Analysis

All bat species were assigned to a dietary niche according to LaVal and Rodríguez [37]: insectivory, phytophagy and carnivory. Phytophagous species included nectarivorous and frugivorous bats, while vampire bats (*Desmodus rotundus*) and species feeding occasionally on vertebrates (*Trachops cirrhosus* and *Phyllostomus hastatus*) were summarised as carnivorous bats. Roosts were categorised after Patterson and colleagues [26]. They established six categories of roost ranks based on logarithmic differences in their estimated durability, with 1 representing the most ephemeral and least protected roost (e.g. rolled leaves and foliage) and 6 the most permanent and protected roost (e.g. caves). As some bat species are known to use different roost types, intermediate ranks were calculated for these species, weighing each roost according to the order in which they were listed in the literature used by Patterson and colleagues (e.g. with three roosts ranked 6, 6, and 5, the weighted rank was calculated as (3×6+2×6+1×5)/6 = 5.83), leading to a continuous scaling of the variable “roost” [26]. Table 1 reports the roost category assigned to each bat species included in this study.

**Table 1:** Mean and standard error of the mean (SEM) for body mass, white blood cell count (WBC) and bacterial killing activity (BKA) of 24 Neotropical bat species.

Species	Body mass (g)		WCB (cells/visual field)			BKA (%)				Roostcategory	Dietaryniche
	Mean	SEM	Mean	SEM	*N*	Mean	SEM	ST	*N*		
*Artibeus jamaicensis*	56.38	2.53	11.59	2.24	13	62.31	6.52	69	11	2	Phytophagy
*Artibeus lituratus*	73.00	0.00	13.10	0.21	2	99.66	0.00	66	1	1.33	Phytophagy
*Artibeus watsoni c.f.*	12.21	0.41	4.60	1.18	7	54.83	7.91	70	8	1	Phytophagy
*Carollia castanea*	13.07	0.20	10.91	2.45	14	85.26	3.07	71	13	4.16	Phytophagy
*Carollia perspicillata*	18.14	0.58	9.89	1.95	11	82.74	5.29	68	12	3.91	Phytophagy
*Carollia sowelli*	16.75	0.58	9.14	2.05	10	94.58	3.34	62	11	4	Phytophagy
*Desmodus rotundus*	39.00	0.41	14.05	1.18	2	74.44	7.91	51	2	4.5	Carnivory
*Ectophylla alba*	5.40	0.06	1.11	0.36	13	86.56	5.32	59	11	1	Phytophagy
*Glossophaga commissarisi*	8.00	0.54	5.78	1.89	9	62.63	5.95	68	9	4.5	Phytophagy
*Glossophaga soricina*	7.82	0.30	3.92	1.44	5	62.57	7.62	62	4	4.66	Phytophagy
*Lophostoma silvicolum*	45.50	4.60	9.90	3.11	2	86.23	0.21	65	2	3	Insectivory
*Mesophylla macconnelli*	8.40	0.07	1.45	0.04	2	86.70	2.02	63	2	1	Phytophagy
*Micronycteris hirsuta*	14.50	0.00	3.90	0.00	1	96.11	0.00	70	1	3	Insectivory
*Micronycteris microtis*	7.00	0.71	1.80	0.42	2	75.49	11.56	67	2	3	Insectivory
*Molossus currentium*	18.83	0.96	2.52	0.44	6	94.46	2.10	52	5	3	Insectivory
*Molossus sinaloe*	26.50	0.00	2.50	0.00	1	43.19	0.00	50	1	4	Insectivory
*Myotis elegans*	5.30	0.00	2.20	0.00	1	86.77	0.00	65	1	2.5	Insectivory
*Phyllostomus discolor*	49.07	3.09	11.21	1.62	7	90.39	5.91	47	7	4	Phytophagy
*Phyllostomus hastatus*	121.13	8.31	14.53	4.40	8	91.68	6.67	65	5	2.6	Carnivory
*Platyrrhinus helleri*	14.67	1.21	3.63	1.04	3	93.48	1.41	57	3	1	Phytophagy
*Rhynchonycteris naso*	4.25	0.18	1.25	0.11	2	100.00	0.00	43	1	2	Insectivory
*Saccopteryx bilineata*	7.89	0.06	3.35	0.52	47	90.18	1.27	61	40	2.37	Insectivory
*Saccopteryx leptura*	5.20	0.14	0.60	0.07	2	87.58	5.25	63	2	3.5	Insectivory
*Trachops cirrhosus*	36.75	2.84	12.25	3.52	4	70.34	3.28	69	4	3.9	Carnivory

White blood cells have been counted on 10 visual fields under 200×magnification with a microscope on a monolayer smear. WBC gives the mean number of cells per visual field. Storage time (ST) of the plasma is given as mean days the samples have been stored at −80°C until assessment of BKA. Diet was drawn from La Val and Rodriguez [37], and species were assigned to three dietary niches: Carnivory (sanguinivorous vampire bat *Desmodus rotundus*, *Trachops cirrhosus* who is specialised on frogs, as well as *Phyllostomus hastatus*, who is omnivorous with a preference for vertebrates [60]), phytophagy (frugivorous and nectarivorous species) and insectivory. Roost category was drawn from Patterson and colleagues [26].

Data on other social and ecological factors such as age, group size and mating system were only available for as small subsample of the species. Therefore, we could not include these factors in our analysis.

**Figure 1:**
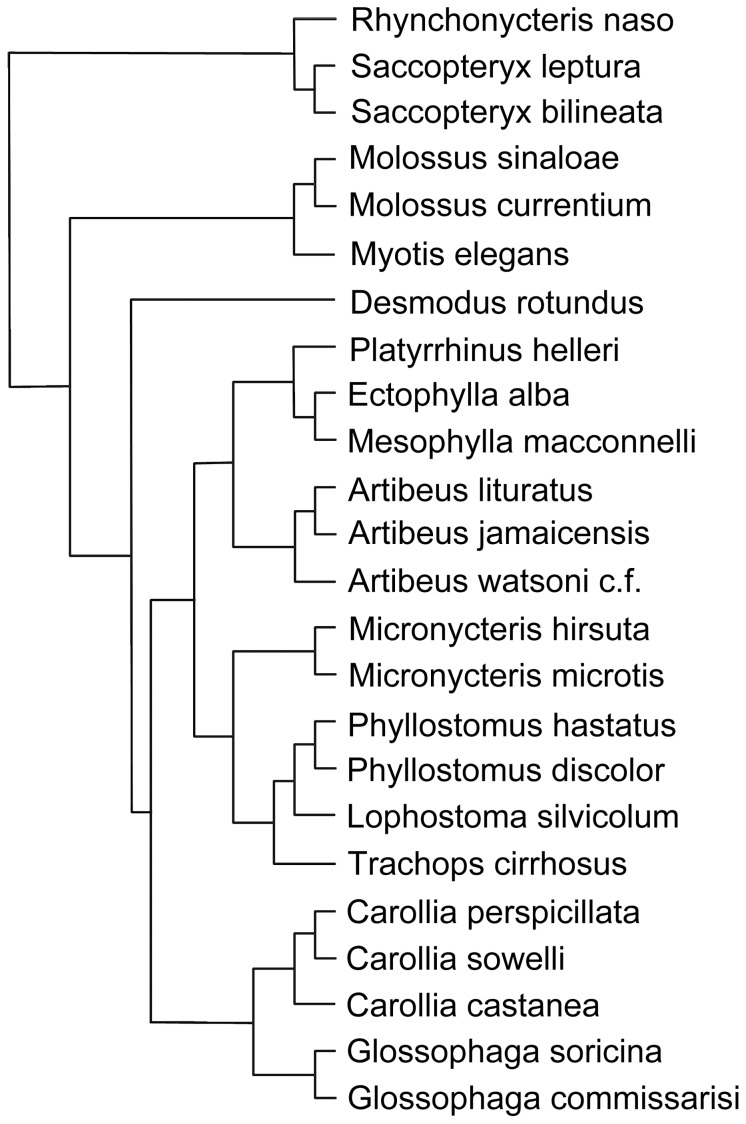
Phylogenetic tree of the 24 Neotropical bat species analysed, modified after Jones and colleagues [**43**]. As it is impossible to distinguish between *Artibeus watsoni* and *Artibeus phaeotis* in the field, we referred to it as *Artibeus watsoni c.f.* For statistical analysis, branch lengths were artificially computed as suggested by Grafen, with the length of a branch being the number of descending taxa minus 1 [45].

We used “R” version 2.13.1 for all statistical analysis [38]. Mean body mass, WBC (total and differential) counts and BKA was calculated for all species. As two closely related species may share inherited characteristics from a common ancestor, species-specific data can not be considered as statistically independent [39]. We therefore calculated phylogenetic generalised least squares models (PGLS [40]) on the effect of dietary niche, roost use and body mass on total and differential WBC count as well as on BKA using the “gls” function of the package “nlme” [41] and accounted for phylogeny using the “correlation” function of the package “ape” [42]. We used a phylogenetic tree modified after Jones and colleagues [43] (Fig. 1). Details of the phylogeny of the genus *Carollia* was drawn from Hoffmann & Baker [44]. We used the covariance matrix “corGrafen” [45], as in initial trials, this resulted in the lowest estimates of model AIC (Akaike’s information criterion). Accordingly, as branch lengths were unknown, we artificially computed them as suggested by Grafen, with the length of a branch being the number of descending taxa minus 1 [45]. As the number of individuals caught for each species varies greatly, we weighted the data by sample size to account for heteroscedasticity of variance. Residuals of the models were normally distributed, except for lymphocytes, where we applied log-transformation. For all analyses, we set the level of significance to α = 0.05.

## Results

We assessed both WBC counts and BKAs in samples from 154 out of 178 captured bats. From the other 24 individuals, we obtained sample volumes that were only sufficient for either WBC or BKA. In 20 out of these 24 individuals, we counted WBCs, and in 4 we measured the BKA. Table 1 reports the mean and standard error of the mean (SEM) for body mass, total WBC counts and BKA as well as the sample sizes for each species. Details on differential WBC counts are given in Table S1 of the electronic supplement.

**Figure 2:**
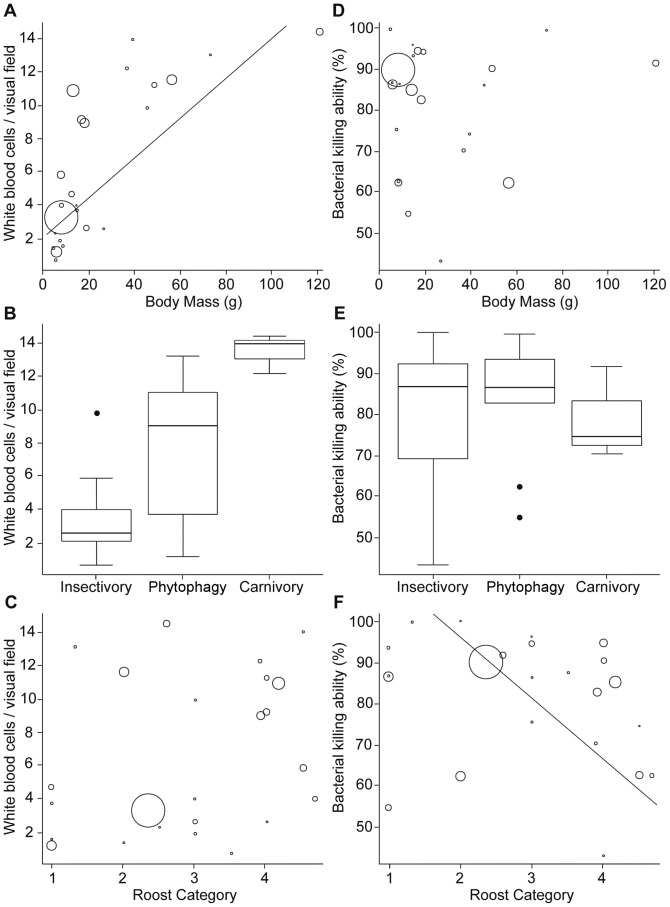
Association between ecological factors and different immune components. Sizes of circles indicate the sample size of each species. Body mass is positively associated with WBC counts, with large species having the highest number of white blood cells (A). WBC count varies among trophic levels (B): Bats feeding on blood and vertebrates (subsumed as carnivorous species) differ significantly in the number of WBCs from insectivorous species, while there is no significant difference between insectivorous and phytophagous bats and only a trend for a difference between phytophagous and carnivorous species. Roost category is not associated with WBC count (C). BKA is not associated with body mass (D). BKA does not differ between dietary niche (E) but decreases with increasing roost permanence and protection (F).

Total WBC counts increased significantly with body mass (t = 5.4; F_1,19_ = 29.2; p<0.001; Fig. 2A), and species differed significantly in their total number of WBCs according to their diet (F_2,19_ = 7.0; p = 0.005; Fig. 2B): Carnivorous bats had significantly higher WBC counts than insectivorous species (t = 3.2; p = 0.005), and phytophagous species had significantly more WBCs than insectivorous species (t = 2.3; p = 0.036). There was no difference between phytophagous and carnivorous bats (t = 1.1; p = 0.27). We found no association between WBC count and roost category (t = 1.6; F_1,19_ = 2.7; p = 0.119; Fig. 2C).

The association of the number of different WBCs with ecological factors depended on the type of WBCs (Table 2). While lymphocytes and basophils were associated with roost permanence and protection, monocytes and eosinophils differed in number between bats with different dietary niches. Carnivorous bats had higher numbers of cells than insectivorous bats (monocytes: t = 2.3; p = 0.030; eosinophils: t = 3.3; p = 0.004). The number of eosinophils were higher in carnivorous than phytophagous bats (t = 2.6; p = 0.017), while there was no significant difference in monocytes (t = 0.9; p = 0.388). Insectivorous and phytophagous species did not differ in the number of cells (monocytes: t = 1.6; p = 0.119; eosinophils: t = 0.21; p = 0.836).

**Table 2:** Association between absolute numbers of different white blood cell types and ecological factors.

**Lymphocytes** (log-transformed)			
**Body mass**	**F_1,19_ = 13.2**	**t = 3.6**	**p = 0.002**
**Roost**	**F_1,19_ = 8.0**	**t = 2.8**	**p = 0.011**
Diet	F_2,19_ = 1.9		p = 0.182
**Monocytes**			
Body mass	F_1,19_ = 0.03	t = 0.17	p = 0.866
Roost	F_1,19_ = 4.2	t = 2.0	p = 0.055
**Diet**	**F_2,19_ = 3.7**		**p = 0.043**
**Neutrophils**			
**Body mass**	**F_1,19_ = 9.0**	**t = 3.5**	**p = 0.008**
Roost	F_1,19_ = 1.3	t = −1.8	p = 0.264
Diet	F_2,19_ = 2.0		p = 0.159
**Basophils**			
**Body mass**	**F_1,19_ = 36.3**	**t = 6.0**	**p<0.001**
**Roost**	**F_1,19_ = 6.0**	**t = **−**2.4**	**p = 0.024**
Diet	F_2,19_ = 0.22		p = 0.804
**Eosinophils**			
Body mass	F_1,19_ = 0.81	t = 0.75	p = 0.381
Roost	F_1,19_ = 0.51	t = −1.4	p = 0.485
**Diet**	**F_2,19_ = 5.5**		**p = 0.013**

Sign of the t-value indicates direction of the correlation. P-values below α = 0.05 are regarded as significant (in bold).

Roost choice of bat species influenced plasma BKA: Species roosting in more ephemeral roosts had lower BKAs that species roosting in permanent roosts (t = −2.8; F_1,19_ = 7.6; p = 0.013; Fig. 2F). There was no significant effect of body mass (t = 0.09; F_1,19_<0.01; p = 0.927; Fig. 2D) or diet (F_2,19_ = 0.29; p = 0.754; Fig. 2E) on BKA.

## Discussion

To our knowledge this is the first inter-specific comparative study investigating ecological factors associated with the immune system in free-ranging mammals. Our study demonstrates that the cellular immune system of bat species varies with diet and body mass, but is not associated with shelter choice, while the soluble part of the constitutive immune function increases with decreasing shelter permanence and protection, but does not vary with body mass and diet.

Based on total WBC counts, we found that the cellular immune system varied among bat species according to their trophic position. We found that total WBC count differed between dietary niches, with bat species preying at least partly on vertebrates or feeding on blood having the highest WBC counts. Carnivorous feeding habits may bear the highest putative risk of acquiring infectious diseases, as parasite and pathogen transmission is expected to be facilitated between more closely related species, i.e. bats and their vertebrate prey. Carnivorous bats apparently invest more in cellular immunity characterised by higher WBC counts in response to the potential threat of becoming infected with vertebrate-specific pathogens. While Nunn and colleagues [16] did not find a general association between carnivory and basal WBC counts, we argue that this might be due to the fact that Nunn and colleagues studied only healthy individuals in captive populations.

Phytophagous bats (frugivorous and nectarivorous species) had an intermediate number of WBCs. Although they do not prey on other vertebrate species, they still may face a notable risk of acquiring pathogens by ingesting contaminated plant matter [10], [22], [23]. Insectivorous bats had the lowest WBC counts. These bats catch prey by flying in open space such as above the canopy or between vegetation. Thus, close contact with potentially contaminated surfaces or infected food is either absent or minimal, and reports about pathogens that are potentially transmitted by ingesting insects are scarce.

Besides the correlation with dietary niche, we found that the cellular immune system of bats was influenced by body mass. This may either simply reflect an allometric relationship, or may have been caused by other factors associated with body mass. Nunn [15] found the number of neutrophils to increase with body mass in primates. He argued that larger primates are more terrestrial than smaller ones, which may lead to body mass being a confounding rather than a causal factor when looking at the influence of ecological factors on the immune system. Vitone and colleagues [10] argued that larger animals also feed on more biomass, which increases the risk of ingesting infectious material. The amount of food ingested by bats can reach up to twice of their body mass (e.g. *Artibeus jamaicensis* [46]), thus large bats may have a higher risk of infestation by parasites and pathogens than small species. Another plausible explanation why large animals generally show a higher WBC counts may be that they are able to host a larger variety of pathogens (‘host as island’ hypothesis [47]). Thus, both diet and body mass are likely to contribute to the evolution of a species cellular immune system. However, there may also be additional factors influencing immunity that were not included in our study, such as physiological stress [48], reproductive status [49], season [50] or infection status.

We found different correlations between dietary niche, roost use and body mass and specific WBC types. While the number of lymphocytes increased with roost permanence and protection, the number of basophils decreased. Lymphocytes are the effectors of the adaptive immune system, having antigen specific cytotoxic (T cells) and secretory (B cells, producing antibodies) roles. Basophils on the other hand release histamines in certain immune responses and thus are important for allergic reactions. However, as the numbers of basophils are usually low (0–13.2% in bats, see ESM), correlations might be susceptible to outliers. Monocytes and eosinophils differed between dietary niches, with carnivorous bats having the highest number of cells, followed by phytophagous and insectivorous species. Monocytes and neutrophils constitute a first line immune defence against invading pathogens, eliminating the intruders via non-specific mechanisms, e.g. phagocytosis. Eosinophils destroy large parasites and are important for modulating allergic inflammatory responses. While the phagocytes respond quickly to a pathogen attack, more time is needed for selection and synthesis of the specific adaptive immune mediators. Thus, species living under higher and more diverse trophic-related risk of infection should rely on a quick non-specific immune defence, and may not invest as much in slower adaptive responses. However, slow-living species such as bats encountering repeated infections are thought to invest in adaptive immunity, while fast-living species should rely on less costly innate immunity due to their investment in growth and early reproduction (‘pace-of-life’ hypothesis [51]). Although white blood cell types have different, sometimes pathogen-specific functions, they are interlinked [51], which makes it difficult to attribute their levels to specific disease or pathogen risks. Thus, a detailed, causative interpretation of DWBC counts at this stage is difficult, but finding associations between DWBC count and ecological factors may potentially reflect the reaction of different cell types on niche-specific risks for pathogen infections.

The soluble part of the constitutive immune system assessed by BKA of the plasma decreased significantly with increasing shelter permanence and protection. A recent study on the bacterial killing ability of whole blood in the Brazilian free-tailed bat (*Tadarida brasiliensis*) [17] demonstrated that BKA may differ among individuals of a species that inhabit different roosting sites. Allen and colleagues argued that differences in ectoparasite abundances of roosts may be causative for differences in the innate immune system of *T. brasiliensis*. In Neotropical bats, ectoparasite load is largely influenced by the roosting behavior of bats: species roosting in more permanent and protected sites are more likely to be infected by parasitic flies and have an overall heavier parasite load [26]. Besides an increased abundance of ectoparasites, well protected shelters may also harbour more vectors for haemoparasites or may promote the accumulation of guano, which may be rich in various bacterial strains, viruses or parasites [52]. However, a higher ectoparasite prevalence found in more permanent shelters may also be caused by the low immunological protections of the animals roosting in such shelters. For example in humans, some pathogens are known to suppress both the cellular and humoral immune response [53]. Although the cause-effect relationship is difficult to assess for the case of bats, our result provides some evidence that the humoral immune system varies among species according to the used type of shelter. Alternatively, species roosting in more ephemeral sites may have to switch roosts more often than species roosting in more permanent sites. Theory predicts that more mobile species may encounter a higher parasite species richness. This theory has found support in mammals [54], birds [55] and fish [56]. For example, it has been found that migratory or dispersing bird species show stronger immune responses than non-migratory and non-dispersing species [57], [58]. Thus, bat species roosting in more ephemeral sites may exhibit an increased BKA compared to species roosting in more permanent sites due to the necessity to change roosts more often and thus potentially coming into contact with more parasites and pathogens when exploring new environments.

Surprisingly, both ecological factors were only associated with one of the two measured aspects of the constitutive immune system, but not the other. Additionally, the effects differed between types of immune cells. Possibly, parasite and pathogen transmission risks vary between ecological factors, including those that might be interlinked with diet and shelter choice but could not be assessed in our study, for example group size, social system, species interactions or human influence. This may ultimately promote only certain domains of the immune system, e.g. cellular versus humoral immune system and adaptive versus innate immunity. While cellular immune functions are energetically costly to mount and maintain [59], humoral aspects are considered to be relatively inexpensive [12]. Thus, investment in different aspects of the immune system may be traded off against each other, depending on the resources an animal is able to invest in overall immunity. However, support for this notion has to come from experimental studies that focus on specific pathogens and the specific immune response of infected animal; a task that is difficult to approach under field conditions.

In conclusion, we provided first evidence from a comparative study that components of the immune system are associated with the ecological factors such as diet and roost use in free-ranging mammals. This implies that certain species are more prone to acquire infectious diseases due to their trophic position or the selection of shelter. Such insights on the effect of ecological factors on immunity and putative disease risk are not only important for conservation, but also to understand potential disease transmission risk and disease dynamics in bats and other mammals.

## Supporting Information

Table S1 **Differential white blood cell counts (absolute and relative mean as well as SEM) of the 24 Neotropical bat species.**
(DOC)
