# Supplementary material for: Measures of the Constitutive Immune System Are Linked to Diet and Roosting Habits of Neotropical Bats
Source: PLoS One. 2013 Jan 14;8(1):e54023. doi: 10.1371/journal.pone.0054023 (PMC3544667; doi:10.1371/journal.pone.0054023)
Supplement: Table S1 — Differential white blood cell counts (absolute and relative mean as well as SEM) of the 24 Neotropical bat species. (DOC) [file pone.0054023.s001.doc]

| **Species** | ***N*** | **Lymphocytes** | **Monocytes** | **Neutrophils** | **Basophils** | **Eosinophils** |
| --- | --- | --- | --- | --- | --- | --- |
| *Artibeus jamaicensis* | 11 |  |  |  |  |  |
| Leucocytes / visual field |  | 9.04 ± 1.88 | 0.03 ± 0.02 | 1.91 ± 0.45 | 0.23 ± 0.13 | 0.39 ± 0.11 |
| % |  | 74.08 ± 2.66 | 0.62 ± 0.26 | 18.54 ± 2.98 | 4.62 ± 1.05 | 2.15 ± 0.91 |
| *Artibeus lituratus* | 1 |  |  |  |  |  |
| Leucocytes / visual field |  | 6.09 ± 2.03 | 0.00 ± 0.00 | 6.49 ± 2.23 | 0.52 ± 0.01 | 0.00 ± 0.00 |
| % |  | 47.00 ± 16.26 | 0.00 ± 0.00 | 49.00 ± 16.26 | 4.00 ± 0.00 | 0.00 ± 0.00 |
| *Artibeus watsoni c.f.* | 8 |  |  |  |  |  |
| Leucocytes / visual field |  | 9.00 ± 2.35 | 0.13 ± 0.06 | 1.17 ± 0.38 | 0.63 ±0.24 | 0.00 ± 0.00 |
| % |  | 78.14 ± 3.84 | 1.57 ± 0.50 | 13.43 ± 2.64 | 6.86 ± 1.61 | 0.00 ± 0.00 |
| *Carollia castanea* | 13 |  |  |  |  |  |
| Leucocytes / visual field |  | 6.88 ± 1.68 | 0.12 ± 0.07 | 1.36 ± 0.41 | 0.60 ± 0.29 | 0.00 ± 0.00 |
| % |  | 71.64 ± 5.56 | 1.91 ± 0.78 | 20.64 ± 5.26 | 5.82 ± 1.86 | 0.00 ± 0.00 |
| *Carollia perspicillata* | 12 |  |  |  |  |  |
| Leucocytes / visual field |  | 6.80 ± 2.01 | 0.10 ± 0.07 | 0.98 ± 0.44 | 0.42 ± 0.11 | 0.01 ± 0.01 |
| % |  | 69.60 ±7.69 | 1.40 ± 0.64 | 21.00 ± 7.77 | 7.40 ± 2.38 | 0.60 ± 0.40 |
| *Carollia sowelli* | 11 |  |  |  |  |  |
| Leucocytes / visual field |  | 2.93 ± 1.14 | 0.21 ± 0.10 | 1.18 ± 0.36 | 0.15 ± 0.09 | 0.12 ± 0.05 |
| % |  | 53.14 ± 8.82 | 4.29 ± 1.78 | 33.14 ± 8.85 | 3.43 ± 1.79 | 6.00 ± 2.49 |
| *Desmodus rotundus* | 2 |  |  |  |  |  |
| Leucocytes / visual field |  | 8.76 ± 1.14 | 0.28 ± 0.10 | 4.72 ± 0.36 | 0.10 ± 0.09 | 0.19 ± 0.05 |
| % |  | 56.00 ± 14.14 | 2.00 ± 0.00 | 39.00 ± 12.02 | 1.00 ± 0.71 | 2.00 ± 1.41 |
| *Ectophylla alba* | 11 |  |  |  |  |  |
| Leucocytes / visual field |  | 0.88 ± 0.33 | 0.02 ± 0.01 | 0.19 ± 0.06 | 0.01 ± 0.01 | 0.01 ± 0.01 |
| % |  | 73.92 ± 4.57 | 0.85 ± 0.40 | 20.62 ± 4.49 | 0.62 ±0.40 | 4.00 ± 3.69 |
| *Glossophaga commissarisi* | 9 |  |  |  |  |  |
| Leucocytes / visual field |  | 4.08 ± 1.65 | 0.02 ± 0.02 | 1.47 ± 0.35 | 0.15 ± 0.07 | 0.07 ± 0.03 |
| % |  | 65.22 ± 6.01 | 0.22 ± 0.21 | 28.44 ± 5.17 | 3.67 ± 1.39 | 2.44 ± 1.12 |
| *Glossophaga soricina* | 4 |  |  |  |  |  |
| Leucocytes / visual field |  | 3.38 ± 1.46 | 0.17 ± 0.11 | 0.28 ± 0.04 | 0.08 ± 0.03 | 0.02 ± 0.01 |
| % |  | 79.00 ± 5.20 | 5.60 ± 3.95 | 12.60 ± 3.37 | 2.00 ± 0.57 | 0.80 ± 0.44 |
| *Lophostoma silvicolum* | 2 |  |  |  |  |  |
| Leucocytes / visual field |  | 8.60 ± 2.82 | 0.10 ± 0.03 | 0.79 ± 0.05 | 0.34 ± 0.16 | 0.07 ± 0.05 |
| % |  | 86.00 ± 1.41 | 1.00 ± 0.00 | 9.50 ± 2.47 | 3.00 ± 0.71 | 0.50 ± 0.35 |
| *Mesophylla macconnelli* | 2 |  |  |  |  |  |
| Leucocytes / visual field |  | 0.91 ± 0.25 | 0.00 ± 0.00 | 0.44 ± 0.14 | 0.04 ± 0.02 | 0.07 ± 0.05 |
| % |  | 62.00 ± 15.56 | 0.00 ± 0.00 | 30.50 ± 10.25 | 2.50 ± 1.77 | 5.00 ± 3.54 |
| *Micronycteris hirsuta* | 1 |  |  |  |  |  |
| Leucocytes / visual field |  | 2.11 ± 0.00 | 0.08 ± 0.00 | 1.56 ± 0.00 | 0.16 ± 0.00 | 0.00 ± 0.00 |
| % |  | 54.00 ± 0.00 | 2.00 ± 0.00 | 40.00 ± 0.00 | 4.00 ± 0.00 | 0.00 ± 0.00 |
| *Micronycteris microtis* | 2 |  |  |  |  |  |
| Leucocytes / visual field |  | 1.07 ± 0.20 | 0.04 ± 0.03 | 0.64 ± 0.23 | 0.02 ± 0.02 | 0.04 ± 0.01 |
| % |  | 61.00 ± 3.54 | 3.00 ± 2.12 | 33.00 ± 4.95 | 1.00 ± 0.71 | 2.00 ± 0.00 |
| *Molossus currentium* | 5 |  |  |  |  |  |
| Leucocytes / visual field |  | 0.91 ± 0.39 | 0.10 ± 0.06 | 1.42 ± 0.48 | 0.03 ± 0.02 | 0.06 ± 0.02 |
| % |  | 35.00 ± 10.95 | 4.33 ± 2.56 | 57.33 ± 13.44 | 1.33 ± 0.61 | 2.00 ± 0.67 |
| *Molossus sinaloe* | 1 |  |  |  |  |  |
| Leucocytes / visual field |  | 2.30 ± 0.00 | 0.00 ± 0.00 | 0.20 ± 0.00 | 0.00 ± 0.00 | 0.00 ± 0.00 |
| % |  | 92.00 ± 0.00 | 0.00 ± 0.00 | 8.00 ± 0.00 | 0.00 ± 0.00 | 0.00 ± 0.00 |
| *Myotis elegans* | 1 |  |  |  |  |  |
| Leucocytes / visual field |  | 0.53 ± 0.00 | 0.00 ± 0.00 | 1.54 ± 0.00 | 0.04 ± 0.00 | 0.09 ± 0.00 |
| % |  | 24.00 ± 0.00 | 0.00 ± 0.00 | 70.00 ± 0.00 | 2.00 ± 0.00 | 4.00 ± 0.00 |
| *Phyllostomus discolor* | 7 |  |  |  |  |  |
| Leucocytes / visual field |  | 7.98 ± 1.62 | 0.30 ± 0.13 | 2.00 ± 0.76 | 0.75 ± 0.20 | 0.19 ± 0.05 |
| % |  | 68.43 ±6.40 | 2.43 ± 0.94 | 20.86 ± 7.76 | 6.71 ± 1.19 | 1.57 ± 0.49 |
| *Phyllostomus hastatus* | 5 |  |  |  |  |  |
| Leucocytes / visual field |  | 8.87 ± 2.65 | 0.18 ± 0.07 | 3.38 ± 1.10 | 1.71 ± 0.56 | 0.38 ±0.16 |
| % |  | 59.88 ± 2.31 | 1.13 ± 0.48 | 23.06 ± 2.57 | 13.19 ± 2.43 | 2.75 ± 0.72 |
| *Platyrrhinus helleri* | 3 |  |  |  |  |  |
| Leucocytes / visual field |  | 2.29 ± 0.93 | 0.04 ± 0.03 | 1.05 ± 0.13 | 0.20 ± 0.08 | 0.05 ± 0.03 |
| % |  | 51.00 ± 16.78 | 0.67 ± 054 | 42.33 ± 17.83 | 4.67 ± 1.09 | 1.33 ± 0.54 |
| *Rhynchonycteris naso* | 1 |  |  |  |  |  |
| Leucocytes / visual field |  | 0.20 ± 0.00 | 0.00 ± 0.00 | 1.05 ± 0.11 | 0.00 ± 0.00 | 0.00 ± 0.00 |
| % |  | 16.00 ± 1.41 | 0.00 ± 0.00 | 84.00 ± 1.41 | 0.00 ± 0.00 | 0.00 ± 0.00 |
| *Saccopteryx bilineata* | 40 |  |  |  |  |  |
| Leucocytes / visual field |  | 2.04 ± 0.38 | 0.03 ± 0.01 | 1.11 ± 0.25 | 0.04 ± 0.01 | 0.00 ± 0.00 |
| % |  | 65.10 ± 3.64 | 1.49 ± 0.33 | 31.46 ± 3.78 | 1.87 ± 0.56 | 0.04 ± 0.03 |
| *Saccopteryx leptura* | 2 |  |  |  |  |  |
| Leucocytes / visual field |  | 0.25 ± 0.14 | 0.00 ± 0.00 | 0.35 ± 0.07 | 0.00 ± 0.00 | 0.00 ± 0.00 |
| % |  | 37.50 ± 19.45 | 0.00 ± 0.00 | 62.50 ± 19.45 | 0.00 ± 0.00 | 0.00 ± 0.00 |
| *Trachops cirrhosus* | 4 |  |  |  |  |  |
| Leucocytes / visual field |  | 9.38 ± 3.84 | 0.05 ± 0.04 | 2.42 ± 1.02 | 0.27 ± 0.09 | 0.13 ± 0.07 |
| % |  | 70.00 ± 12.39 | 0.50 ± 0.43 | 25.50 ± 12.40 | 2.50 ± 1.09 | 1.50 ± 0.83 |
